# Development of an Improved Stiffness Ellipsoid Method for Precise Robot-Positioner Collaborative Control in Friction Stir Welding

**DOI:** 10.3390/ma18081852

**Published:** 2025-04-17

**Authors:** Cunfeng Kang, Haonan Jia, Eryang Zhao, Chunmin Ma

**Affiliations:** College of Mechanical and Energy Engineering, Beijing University of Technology, Beijing 100124, China; 17325533164@163.com (H.J.); m15856492704@163.com (E.Z.); machunmin@bjut.edu.cn (C.M.)

**Keywords:** friction stir welding robots, stiffness ellipsoid method, collaborative motion control, stiffness index, industrial robotic welding

## Abstract

This study proposes an improved stiffness ellipsoid method to enhance the stiffness and precision of robotic arms in friction stir welding (FSW) operations. The method involves establishing a joint stiffness model through static identification experiments and developing a novel stiffness index derived from the improved stiffness ellipsoid method. This index provides a refined metric for evaluating the robot’s performance under variable loads during FSW. Simulation experiments demonstrate significant improvements in welding trajectory precision and computational efficiency. The findings highlight the potential of this method to elevate FW quality and consistency.

## 1. Introduction

Friction stir welding (FSW) has emerged as a revolutionary technique in modern manufacturing, particularly for producing high-strength, defect-free welds in materials that are challenging to join using conventional methods [1]. This technique, which utilizes a non-consumable rotating tool to join metal pieces, has gained significant attention due to its numerous advantages, including high welding quality, low heat input, and minimal distortion [2]. As a result, FSW has been increasingly integrated with industrial robots to enhance the precision and efficiency of welding operations, making it a critical process in various industries such as automotive, aerospace, and shipbuilding.

However, a persistent and significant challenge in robotic FSW is maintaining precise alignment between the actual welding trajectory and the theoretical path [3]. This misalignment primarily arises from the dynamic interactions between the robot, the welding tool, and the workpiece, which can lead to inconsistencies in joint stiffness and external forces, causing significant deviations and affecting the overall quality of the weld [4,5]. These deviations not only compromise the structural integrity of the weld but also increase production costs due to rework and inspection requirements.

In the realm of industrial robotics, several studies have attempted to address these challenges. For instance, a pioneering study introduced a method to adjust the end-effector stiffness of industrial robots through the shaping of stiffness ellipsoids [6]. This approach, which requires fewer parameters, utilizes a Sequential Least Squares Programming algorithm to determine the optimal joint positions and stiffness values, showing significant improvements in ellipsoid shaping in simulated environments. However, existing methods generally lack a sufficiently refined metric for evaluating the performance of robots under variable loads during the FSW process.

Given these limitations, there is a clear need for an improved method that can provide a more accurate and comprehensive assessment of robotic performance in FSW operations. This study proposes an improved stiffness ellipsoid method to enhance the stiffness and precision of robotic arms in FSW. By establishing a joint stiffness model through static identification experiments and developing a novel stiffness index derived from the improved stiffness ellipsoid method, this study aims to fill the gap in the current literature and provide a more effective control strategy for industrial applications. The proposed method not only improves the precision of the welding trajectory but also enhances computational efficiency, making it a promising solution for practical industrial use [7,8,9,10,11,12,13].

## 2. Materials and Methods

### 2.1. Robot Joint and End-Effector Stiffness Modeling

The establishment of a joint stiffness model first requires the stiffness of each joint of the robot, and then, based on the mapping relationship between the robot joint stiffness and the Cartesian stiffness of the end effector, the Cartesian stiffness model of the robot end effector in this posture can be obtained. This study used the static identification experimental method to obtain the joint stiffness of the robot. In the static state of the robot, an external force was loaded at the end, and the load was measured using a six dimensional force sensor. Based on the relationship between the external load and torque of the welded joint, the stiffness of each joint was calculated, and a joint stiffness model of the robot could be established, followed by a Cartesian stiffness model of the end of the robot. 

The stiffness identification experiment was conducted to measure the joint stiffness of the robot. A six-dimensional force sensor was used to measure the forces acting on the robot’s end. By applying an external force at the robot’s end and measuring the resulting deformations, the joint stiffness model was established. The experiment involved loading an external force at the end of the robot and measuring the load with a six-dimensional force sensor. Based on the relationship between the external load and joint torque, the stiffness of each joint was calculated, and a joint stiffness model of the robot was established [14]. The stiffness identification model for the robot establishes the relationship between the end effector force and joint deformation, solving the joint stiffness of the robot and then establishing the joint stiffness model and end effector Cartesian stiffness model of the robot. The mapping relationship between the robot joint stiffness model and the end Cartesian stiffness model was analyzed to derive the stiffness characteristics of robot’s end [15].

The joint stiffness values listed in Table 1 serve as a reference for the base configuration of the ZK3000-500 robot in order to derive new stiffness index through experimental validation. The joint stiffness of the robot was previously identified, and the joint stiffness model of the robot was established. Table 1 lists the joint stiffness values of the ZK3000-500 robot. These stiffness values were obtained through static identification experiments and are used to establish the robot’s joint stiffness model. Each joint stiffness value reflects the joint’s ability to resist deformation when subjected to external forces. The joint stiffness values are as follows:

### 2.2. Enhancing Robotic Friction Stir Welding Through Stiffness Ellipsoid Modeling

In the friction stir welding (FSW) process, the robot end experiences significant forces, primarily from the forging pressure in the normal direction of the weld seam. To ensure high-precision weld seam tracking and quality, it is crucial to develop a new stiffness index that accounts for the robot’s dynamic interaction with the work piece. This study introduces a novel stiffness index derived from an improved stiffness ellipsoid model, which provides a more comprehensive assessment of the robot’s performance during FSW operations. To substantiate the effectiveness of the new stiffness index, a series of experiments were conducted using a ZK3000-500 robot. The robot’s end effector was subjected to controlled forces, imitating the conditions of FSW, and the resulting deformations were measured. The data collected were then used to calculate the new stiffness index and compare it with the conventional stiffness assessments [16,17]. 

**Figure 1:**
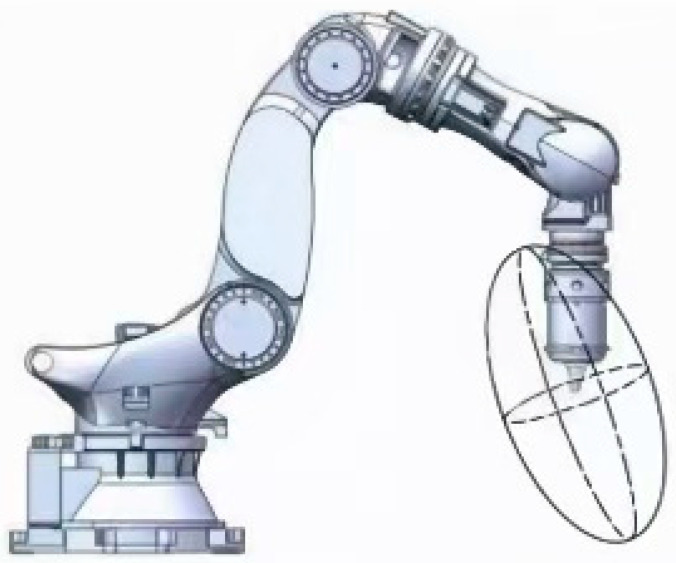
Robot directional stiffness index.

The lengths of the semi-axes of the ellipsoid are the inverse square roots of the eigenvalues of the flexibility matrix, which can reflect the characteristics of the robot’s end stiffness, commonly referred to as the robot’s Cartesian stiffness ellipsoid. The shortest semi-axis represents the direction of the minimum stiffness of the robot’s end at the current position, while the longest semi-axis represents the direction of the maximum stiffness. A greater difference between the longest and shortest semi-axes of the ellipsoid would result in a greater difference in stiffness in different directions at the current posture of the robot’s end, and vice versa, the stiffness distribution of the robot’s end is more uniform. Therefore, the volume of the stiffness ellipsoid can be used to evaluate the overall stiffness of the robot’s end at a certain posture; a larger volume would lead to more uniform stiffness distribution and better overall stiffness, as shown in Figure 1.

The experimental setup for joint stiffness identification is shown in Figure 1. The ZK3000-500 robot was used as the test platform, with its end-effector mounted with a high-precision six-dimensional force sensor (ATI Nano17, with a force measurement range of ±17 N and a torque measurement range of ±1.7 Nm). The force sensor is capable of measuring forces and torques in six directions (Fx, Fy, Fz, Tx, Ty, Tz) with high accuracy and resolution. The robot was positioned in a static state, and the end-effector was fixed at a predetermined position to ensure stability during the experiment.

Calibration of the Force Sensor: Before conducting the stiffness identification experiments, the six-dimensional force sensor was calibrated according to the manufacturer’s instructions. The calibration process involved applying known forces and torques to the sensor and recording the corresponding output signals. The calibration data were used to establish a linear relationship between the sensor output and the applied forces/torques, ensuring accurate measurements.

The robot was placed in a static state with its end-effector positioned at a fixed point in space. External forces were applied to the end-effector in multiple directions (Fx, Fy, Fz) using a calibrated force applicator. The forces were applied incrementally, with each increment held for a sufficient duration to allow the system to reach equilibrium. The six-dimensional force sensor measured the applied forces and torques, and the corresponding joint torques were recorded using the robot’s control system. The collected data were processed using MATLAB version 2020a to derive the stiffness characteristics of each joint. The stiffness values were calculated based on the relationship between the external forces and the resulting joint torques. The joint stiffness model was established by fitting the calculated stiffness values to a mathematical model that describes the stiffness behavior of the robot’s joints. The model was validated by comparing the predicted stiffness values with the measured data obtained from the experiments. The validation process involved multiple iterations and cross-checks to ensure the accuracy and reliability of the model.

### 2.3. Analysis of Robot Stiffness Characteristics

**Figure 2:**
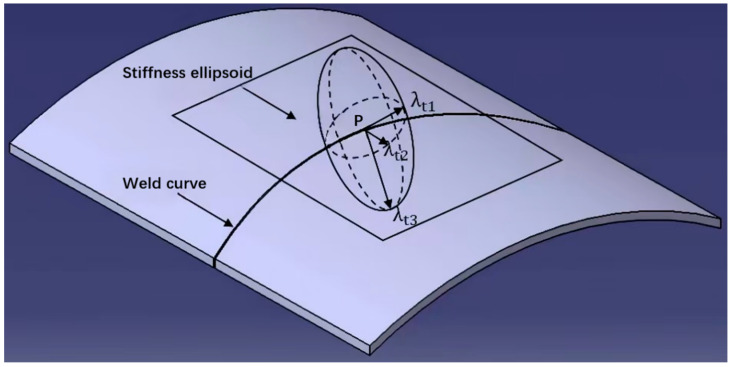
Stiffness ellipsoid in welding surfaces.

The lengths of the semi-axes of the ellipsoid are the inverse square roots of the eigenvalues of the flexibility matrix, which can reflect the characteristics of the robot’s end stiffness, commonly referred to as the robot’s Cartesian stiffness ellipsoid. The shortest semi-axis represents the direction of the minimum stiffness of the robot’s end at the current position, while the longest semi-axis represents the direction of the maximum stiffness. A greater difference between the longest and shortest semi-axes of the ellipsoid would result in a greater difference in stiffness in different directions at the current posture of the robot’s end, and vice versa, the stiffness distribution of the robot’s end is more uniform. Therefore, the volume of the stiffness ellipsoid can be used to evaluate the overall stiffness of the robot’s end at a certain posture; the larger volume would lead to more uniform stiffness distribution and better overall stiffness, as shown in Figure 2. According to the mapping relationship between the robot joint stiffness model and the end Cartesian stiffness model, the Cartesian stiffness matrix of the robot end was calculated as follows:

(1) $$K={\left(J^{-1}\right)}^{T}\cdot K_{\theta }\cdot J^{-1}=\left[\begin{array}{cccc} k_{11} & k_{12} & \cdots & k_{16}\\ k_{21} & k_{22} & \cdots & k_{26}\\ \vdots & \vdots & \ddots & \vdots \\ k_{61} & k_{52} & \cdots & k_{66} \end{array}\right]$$

The specific content of the formula can be retrieved from Table 2. The elements of the Cartesian stiffness matrix was divided into different blocks according to their physical meanings:

(2) $$\left[\begin{array}{c} F\\ M \end{array}\right]=\left[\begin{array}{cc} K_{fd} & K_{f\delta }\\ K_{md} & K_{m\delta } \end{array}\right]\left[\begin{array}{l} d\\ \delta \end{array}\right]$$

where $K_{fd},K_{fd},K_{md},K_{m\delta }$ are stiffness sub matrixs of 3×3, and $K_{fd}$ represents the sub matrix of the force displacement stiffness; $K_{fd}$ represents the sub matrix of force angular displacement stiffness; $K_{md}$ represents the sub matrix of torque displacement stiffness; $K_{m\delta }$ represents the sub matrix of torque angular displacement stiffness; d represents the linear displacement of the robot’s end along the X,Y, and Z axes; δ represents the angular displacement of the robot end around the X,Y, and Z axes. Combining the relationship between the end force and deformation displacement of the FSW robot, when establishing the stiffness ellipsoid, only the $K_{fd}$ stiffness sub matrix of the Cartesian stiffness matrix was considered. Therefore, the relationship between the end force of the robot and the end line displacement was written as follows:

(3) $$F_{d}=K_{fd}\cdot d=\left[\begin{array}{ccc} k_{11} & k_{12} & k_{13}\\ k_{21} & k_{22} & k_{23}\\ k_{31} & k_{32} & k_{33} \end{array}\right]\cdot d$$

The Cartesian stiffness matrix of the robot end effector has symmetry, and thus the stiffness sub matrix $K_{fd}$ composed of its first 3 rows and first 3 columns elements clearly also has symmetry. Therefore, the expression for the ellipsoid of the robot end effector stiffness is as follows:

(4) $$\frac{x^{2}}{\lambda_{1}}+\frac{y^{2}}{\lambda_{2}}+\frac{z^{2}}{\lambda_{3}}=1$$

In the formula, x,y, and z represent the origin coordinates of the stiffness ellipsoid and also represent the coordinates of the robot end effector, indicating that the stiffness ellipsoid was attached to the robot end effector; $\lambda_{1},\lambda_{2},\lambda_{3}$ represent the length of three half axes perpendicular to each other along the x,y, and z axes of the stiffness ellipse, which were obtained from the eigenvalues of the real symmetric matrix $K_{fd}^{T}\cdot K_{fd}$. The eigenvector $\eta_{1},\eta_{2},\eta_{2}$ of the real symmetric matrix represent the vector direction of the corresponding half axis of the eigenvalues.

In FSW, although there is a certain welding angle between the stirring head and the weld seam during the welding process, this angle is usually small. Therefore, when studying the stiffness characteristics of robot welding, the axis of the stirring head is basically along the normal direction of the weld seam, which is perpendicular to the tangent plane of the welding surface. The forward resistance and lateral force during welding are both within the tangent plane and perpendicular to each other, and the forging force is perpendicular to the tangent plane. Therefore, the stiffness ellipse needs to be mapped to the tangent plane of the welding surface in order to be effective. Assuming that at a certain moment during welding, the stirring head at the end of the robot coincides with the welding point in the weld seam, the origin of the stiffness ellipsoid at the end of the robot should also coincide with the welding point. Figure 2 shows that the stiffness ellipsoid intersects with the tangent plane of the welding surface [18,19,20]. 

While the improved stiffness ellipsoid method offers significant benefits in terms of enhanced welding trajectory precision and computational efficiency, it also presents some practical limitations in industrial applications. One major limitation is the real-time adaptability of the method. The current implementation relies on pre-calculated stiffness models and optimized trajectories, which may not be fully adaptive to dynamic changes in the welding environment, such as variations in material properties or unexpected external forces. Additionally, the method requires precise calibration and setup, which can be time-consuming, and may need periodic recalibration to maintain accuracy. To address these challenges, several strategies can be considered. First, integrating advanced sensors, such as force/torque sensors and vision systems, can provide real-time feedback on the welding process, enabling dynamic adjustments to the stiffness model and trajectory planning. Second, developing adaptive control algorithms that can learn and adjust to variations in the welding conditions can enhance the robustness of the method. Finally, implementing a modular and flexible calibration process can reduce setup time and improve the ease of use in industrial settings.

The specific content of the formula can be retrieved from Table 2. The analytical formula for the stiffness ellipse is as follows:

(5) $$\left\{\begin{array}{c} \frac{x^{2}}{\lambda_{1}}+\frac{y^{2}}{\lambda_{2}}+\frac{z^{2}}{\lambda_{3}}=1\\ xcos\alpha_{t}+ycos\beta_{t}+zcos\gamma_{t}=0 \end{array}\right.$$

where $cos\alpha_{t},cos\beta_{t},cos\gamma_{t}$ respectively represent the direction cosine of the unit normal vector on the tangent plane. The long half axis of the ellipse is $\lambda_{t1}$ and the short half axis is $\lambda_{t2}$, and the distance from the tangent plane normal to the surface of the stiffness ellipsoid was determined as $\lambda_{t3}$ in this case. The physical meanings of $\lambda_{t1},\lambda_{t2},\lambda_{t3}$ are the stiffness magnitude in their respective directions.

The basic method for constructing a new stiffness index is to use the stiffness in the $\lambda_{t1}$ direction to resist the forward resistance during friction stir welding, use the stiffness in the $\lambda_{t2}$ direction to resist the lateral force during welding, and use the stiffness in the $\lambda_{t3}$ direction to resist the normal force during welding. The previous studies on friction stir welding have shown that the forward resistance during the friction welding process is approximately 0.75 times that of the normal force, and the welding lateral force is usually small. The value of $\lambda_{t2}$ can depend on the existence of $\lambda_{t1}$. A new stiffness index based on an improved stiffness ellipsoid was constructed in the form of an evaluation function, and the new stiffness is shown as follows:

(6) $$V_{f}=\sqrt{\lambda_{t1}^{2}+\lambda_{t3}^{2}}\cdot e^{-\left|\frac{\lambda_{t1}}{\lambda_{t3}}-\frac{3}{4}\right|}$$

The specific content of the formula can be retrieved from Table 2. The evaluation function of this new stiffness index is initially non-negative, which is beneficial for solving the collaborative control model of robots and displacement machines in the future. This indicator represents the size and direction of the end stiffness in the tangent plane of the welding surface during the robot welding process using the values of $\lambda_{t1}$ and $\lambda_{t3}$, taking into account the ratio between the values of $\lambda_{t1}$ and $\lambda_{t3}$, which is suitable for the requirements of friction stir welding technology.

### 2.4. Composition and Kinematic Modeling of the Robot Friction Stir Welding System

**Figure 3:**
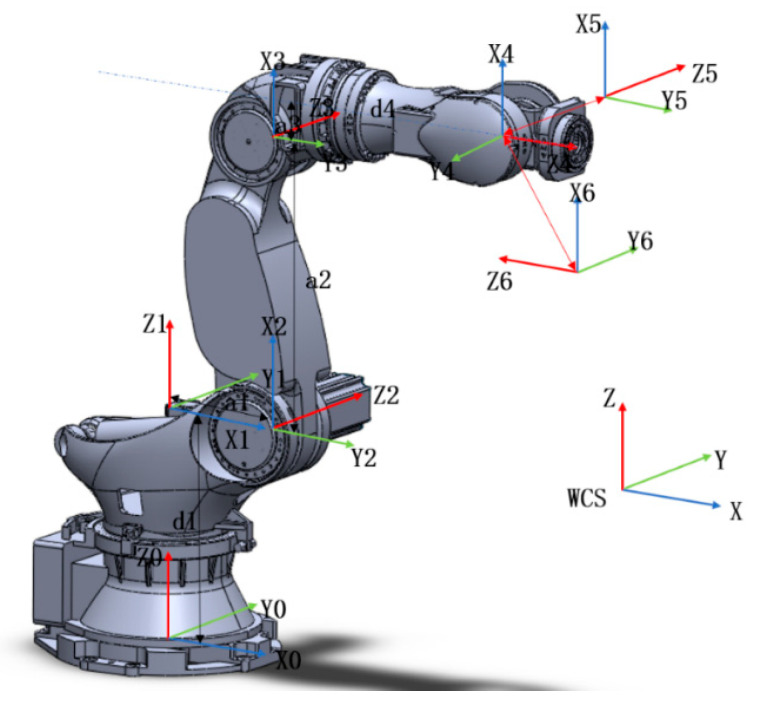
D-H coordinate system of the ZK3000-500 robot.

To establish the kinematic model of the robot and the positioning machine, it is necessary to first establish the coordinate systems to describe the spatial relative position and attitude of each joint and link with respect to the base coordinate system [21]. Based on the known link parameters, an improved Denavit–Hartenberg (D-H) method was used to establish the coordinate system of each joint link, obtaining the welding parameters such as link length, link offset, link angle, and joint angle (Figure 3). 

### 2.5. Collaborative Motion Control Algorithm for Robot and Positioner Based on the Stiffness Index

#### 2.5.1. Collaborative Motion Control Model of Robot and Positioner

The goal of the collaborative motion control model for friction stir welding is to use the weld seam information and other necessary welding process parameters of the welded part as inputs to the system, solve for the joint angles of the robot and the positioner in collaborative motion, and select the optimal joint angle combination based on the new stiffness index to control the motion of each weld joint of the robot and the positioner.

A mathematical model for a collaborative control method was established based on a new stiffness index. the control model of the friction stir welding robot and the displacement machine system were divided into the objective functions, decision variables, and constraint conditions. The specific content of the formula can be retrieved from Table 2.

(1) Objective function

We set the new stiffness index evaluation function proposed in the previous section as the objective function of the collaborative motion control model. The process of solving the collaborative motion control model is the process of finding the maximum objective function. The setting of the objective function is as follows:

(7) $$V_{m}=max\left(V_{f}\right)=max\left(\sqrt{\lambda_{t1}^{2}+\lambda_{t3}^{2}}\cdot e^{-\left|\frac{\lambda_{t1}}{\lambda_{t3}}-\frac{3}{4}\right|}\right)$$

(2) Decision variables

The decision variables are related to the eight joint angles of the collaborative motion system. The strategy for collaborative motion is to obtain the position and posture information of the welded joint points of the work piece, and then the two degree of freedom displacement machine performs calculation of the active motion and forward kinematics; the six axis heavy-duty industrial robot performs passive motion, and the calculation result of the robots six joint angles is a function of the joint angles $\theta_{P1}$ and $\theta_{P2}$ of the displacement machine. The decision variables of the collaborative motion control model are represented as a function of the robot joint angle with respect to the displacement machine joint angle which is shown as follows:

(8) $$\theta =f\left(\theta_{P1},\theta_{P2}\right)\cdot T_{n}$$

where $T_{n}$ represents the pose information of the weld joint in the robot base coordinate system and $\theta =f\left(\theta_{P1},\theta_{P2}\right)\cdot T_{n}\;$represents the functional relationship between the joint angle of the positioner and the robot after pose transformation of the weld joint.

(3) Constraints

We limit the motion of the robot in the decision variable, and the joint angle of the robot’s motion should not exceed its joint limit; the joint movement of the displacement machine should also not exceed its joint limit, and we ensure that the welding point position on its workbench does not exceed the robot’s workspace after the displacement machine has been moved.

Finally, the indicator-based collaborative motion control model for robots and displacement machines is summarized as follows:

(9) $$\left\{\begin{array}{c} V_{m}=max\left(V_{f}\right)\\ \theta =f\left(\theta_{P1},\theta_{P2}\right)\cdot T_{n}\\ min\theta \leq \theta \leq max\theta \\ min\theta_{P}\leq \theta_{P}\leq max\theta_{P}\\ T_{n}\in W \end{array}\right.$$

#### 2.5.2. Solution of Collaborative Motion Control Model

(1) Genetic algorithm for solving the model

To solve the collaborative motion control model of this project using a genetic algorithm, modifications need to be made to the model to meet the format requirements of the genetic algorithm. The specific settings are as follows:

a. Fitness function settings

We use a new stiffness index $V_{f}$ based on stiffness ellipsoids as the fitness function. The size of the new stiffness index is related to the joint angles of the robot and positioner; therefore, the fitness function is $S\left(\theta \right)$, and its expression is as follows:

(10) $$S\left(\theta \right)=max\left(V_{f}\left(\theta \right)\right)$$

b. Design variables

The variables of the collaborative motion control model are the two joint angles of the displacement machine and the six joint angles of the robot. The robot joint angle is a function of the displacement machine joint angles $\theta_{P1}$ and $\theta_{P1}$. We set the design variable to the joint angle of the positioner:

(11) $$\theta_{p}={\left[\begin{array}{cc} \theta_{P1} & \theta_{P2} \end{array}\right]}^{T}$$

The joint angle of the robot is as follows:

(12) $$\theta =f\left(\theta_{P}\right)\cdot T_{n}$$

c. Boundary condition settings

The boundary conditions of the system can be considered as constraints on the motion of robots and displacement machines. The boundary conditions are summarized as follows:

(13) $$\left\{\begin{array}{c} 0\leq T\in W\\ \theta \in \left[\theta_{min}\;\theta_{max}\right]\\ \theta_{P}\in \left[\theta_{P_{min}}\;\theta_{P_{max}}\right] \end{array}\right.$$

d. Initial population generation

When the robot displacement machine system cooperates in motion, for any welding interpolation point, 50 sets of different joint angles were randomly selected within the motion range of the two rotation axes of the displacement machine:

(14) $$\theta_{P}=\left[\begin{array}{cc} \theta_{P1i} & \theta_{P2i} \end{array}\right]\left(i=1,\cdots ,50\right)$$

Compile 50 sets of joints into 50 chromosomes using binary encoding as the initial population.

e. Selection method and crossover operator

Using rthe oulette wheel selection method to select individuals in the population in order to quickly eliminate low fitness individuals, the crossover method is uniform crossover and crossover operator $p_{c}=0.8$.

f. Algorithm termination condition

Taking the achievement of the target genetic algebra as the termination condition of the genetic algorithm, and taking into account the real-time operation and accuracy requirements of the algorithm, this paper sets the number of genetic iterations to g = 100. When g > 100, the optimization process stops.

(2) Improvement of the Genetic Algorithm

In order to improve the success rate and efficiency of genetic algorithm solving, the generation method of mutated individuals in genetic algorithm was improved as follows: after the initial population of mutated machine joint angles is generated, a series of robot postures that meet cooperative motion was obtained. For these robot postures, the position information of the solutions that meet the constraint conditions was kept unchanged, and the direction of the attitude vector was rotated within a small angle. In light of the minute modification in the rotational attitude, the rotated entities demonstrate the capacity to satisfy the constraint conditions. By solving the angle of the displacement machine through collaborative motion, the objective function, i.e., the new stiffness index value, was calculated during this process.

Assume that the pose point of a robot end point that satisfies the constraint conditions is $T_{n}$, and the pose vector represented by this pose point is $R_{n}$. To reduce the computational complexity, the rotation of the vector was simplified as rotating around three coordinate axes. The rotated robot end pose vector is as follows::

(15) $$V_{n}=R_{n}\cdot Rot\left(X,\alpha_{n}\right)\cdot Rot\left(Y,\beta_{n}\right)\cdot Rot\left(Z,\gamma_{n}\right)$$

where $\alpha_{n},\beta_{n},\gamma_{n}$ are a series of angle values for the rotation of the attitude vector. In order to ensure that the motion range of the robot and the displacement machine does not exceed the constraint conditions, for the attitude vector of any point, each rotation around the coordinate axis is $0.01^{\circ }$, and the rotation range does not exceed $1^{\circ }$. The specific content of the formula can be retrieved from Table 2.

## 3. Discussion

### 3.1. Collaborative Motion Control Simulation Experiment

**Figure 4:**
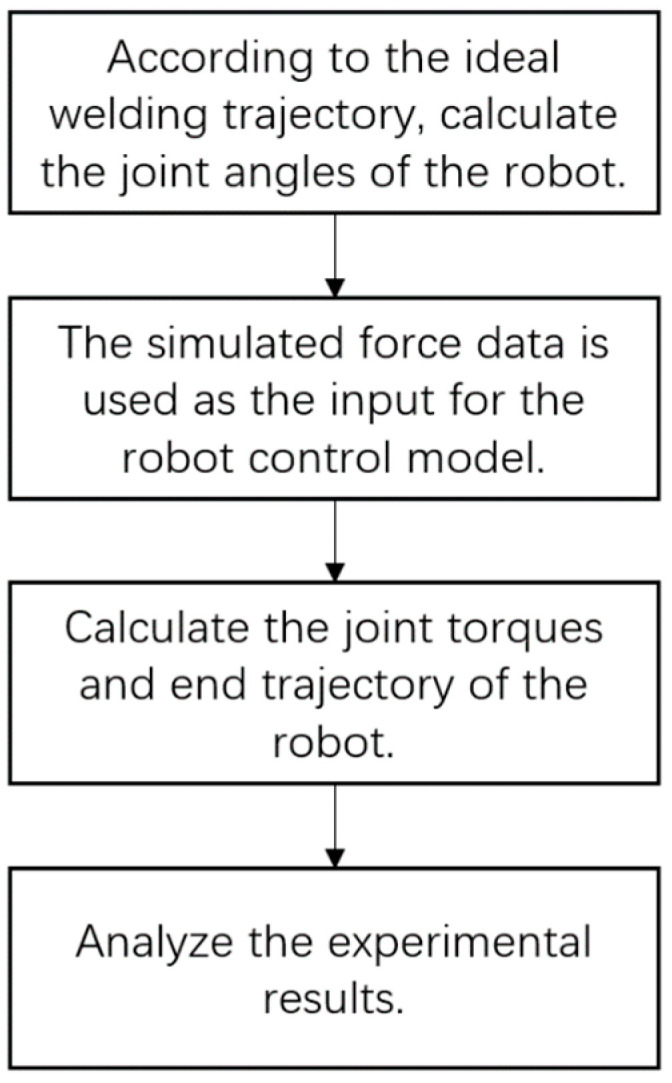
Flow chart of the control simulation experiment.

This trajectory is then used to compute the joint angle data required for the robot during welding, leveraging the robot’s inverse kinematics model. Subsequently, force data, derived from the simulation at the mixing head’s end, is fed into the Simulink model for the robot’s force-position hybrid control via an interface. This force data serves as the input for the robot’s end force, enabling the calculation of the desired joint torques necessary to achieve the optimal end force. The experiment culminates in a comparison between the actual robot end trajectory during the welding process and the ideal welding trajectory, with the analysis of the experimental results focusing on the correlation between the robot end force and the input force. This approach integrates the improved stiffness ellipsoid method to enhance the robot’s performance, ensuring that the actual welding trajectory closely aligns with the theoretical path, which is critical for maintaining the quality and precision of the friction stir welding process. The process of the simulation experiment for robot friction stir welding control is shown in Figure 4.

By integrating the improved stiffness ellipsoid method, we account for the robot’s varying stiffness across different poses, ensuring that the selected pose optimizes the robot’s overall stiffness. The stiffness invariance principle is applied to ensure that the stiffness evaluation remains consistent across the different coordinate systems, which is essential for maintaining the precision and quality of the welding process. This comprehensive approach allows for a more accurate assessment of the robot’s performance under the variable loads during the friction stir welding process, leading to better tracking and enhanced quality of the weld seam.

### 3.2. Welding Pose Optimization Calculation Example

Using the improved genetic algorithm as an example, the collaborative motion pose of the robot and the positioner during welding was optimized. This optimization process leverages the stiffness ellipsoid method to evaluate the robot’s stiffness performance in various poses, ensuring that the selected pose maximizes the robot’s overall stiffness. The stiffness invariance principle was applied to ensure that the stiffness evaluation is consistent across different coordinate systems, which is crucial for maintaining the precision and quality of the welding process. The values of the angles of each axis of the positioner and the robot, as well as the corresponding new stiffness evaluation index before and after optimization, were obtained, as shown in Table 3. This table presents a comprehensive analysis of the robot’s stiffness characteristics in different poses, highlighting the importance of selecting an optimal pose that enhances the robot’s stiffness and minimizes deformation during the welding process. The optimization process began with the identification of the robot’s joint stiffness through the static identification experiments, which established a joint stiffness model. This model is essential for predicting the robot’s deformation under load and forms the basis for proposing a new stiffness index. The new stiffness index, derived from the improved stiffness ellipsoid method, providing a nuanced metric for evaluating the robot’s performance under variable loads during FSW operations.

By employing the improved genetic algorithm, we can find the optimal pose that maximizes the new stiffness index, ensuring that the robot’s motion is synchronized with the positioner throughout the friction stir welding process. This synchronization was critical for maintaining the quality and consistency of the weld, as it reduces the impact of external forces and minimizes the risk of deviation from the theoretical welding path. The results, as depicted in Table 2, demonstrate the effectiveness of the optimization process in improving the robot’s stiffness performance. The before and after optimization values of the stiffness evaluation index illustrate the significant enhancement of the robot’s stiffness, which directly contributes to the precision and quality of the weld joint [22].

**Figure 5:**
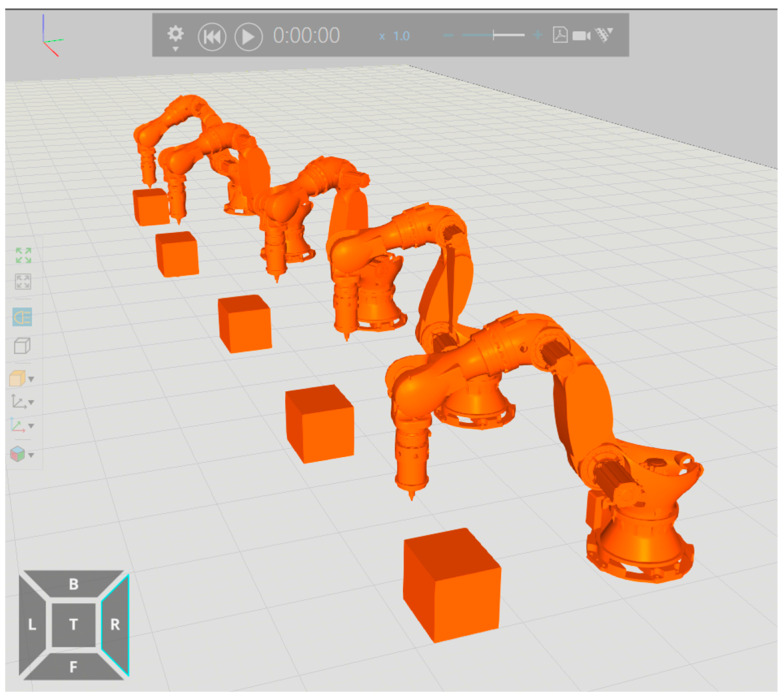
Posture of robot and positioner before and after optimization.

Figure 5 illustrates the significant pose changes of the robot and positioner before and after optimization using different welding spots. To achieve these optimizations, the ZK-500 robot model was imported into Visual Components software 4.7 for comprehensive trajectory planning. This software facilitated the generation of collision-free programs and optimized processes, ensuring that the robot’s movements were precise and efficient throughout the welding process.

### 3.3. Welding Simulation Experiment

**Figure 6:**
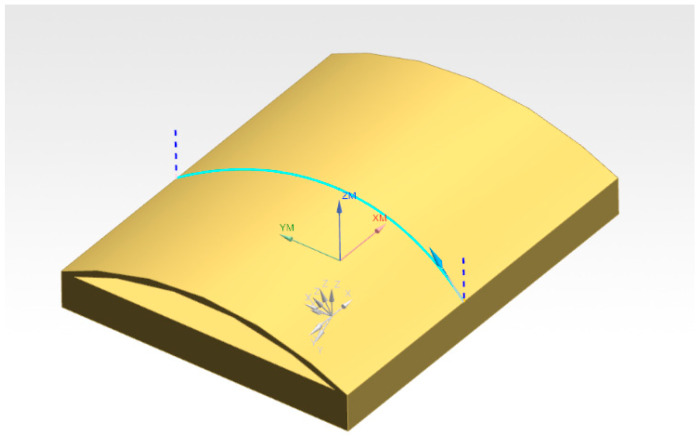
Welded parts and weld trajectory.

**Figure 7:**
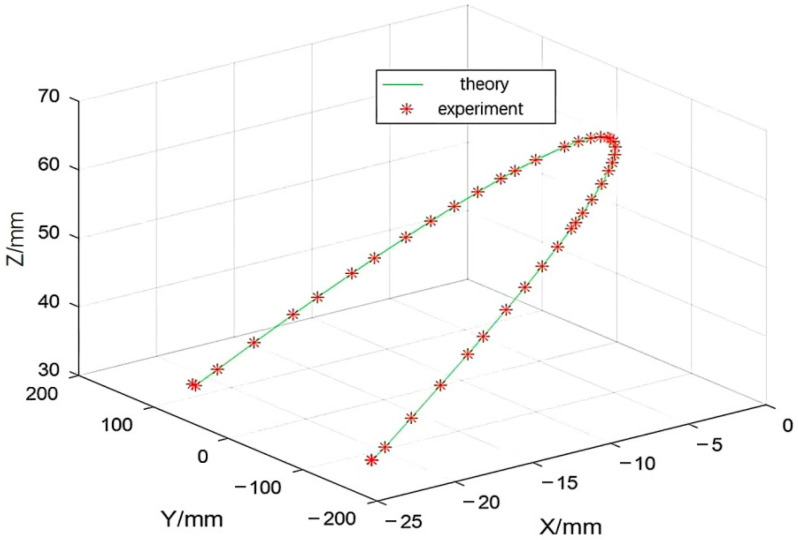
Comparison of weld trajectory.

In NX CAM software version 12.0, the weld seam trajectory was automatically generated for the welded part, which is a spatial curve weld seam, to verify the control effect of the robot and displacement machine collaborative control algorithm on the continuous trajectory. Figure 6 shows the welded part and weld seam trajectory:

Figure 7 shows that the weld trajectory obtained from the collaborative motion control simulation experiment was compared with the ideal weld trajectory. The experimental weld trajectory was basically consistent with the ideal weld trajectory, showing good tracking performance. The trajectory of the robot and the positioner in the welding simulation experiment can verify the effectiveness and accuracy of the collaborative motion control algorithm based on the stiffness index.

**Figure 8:**
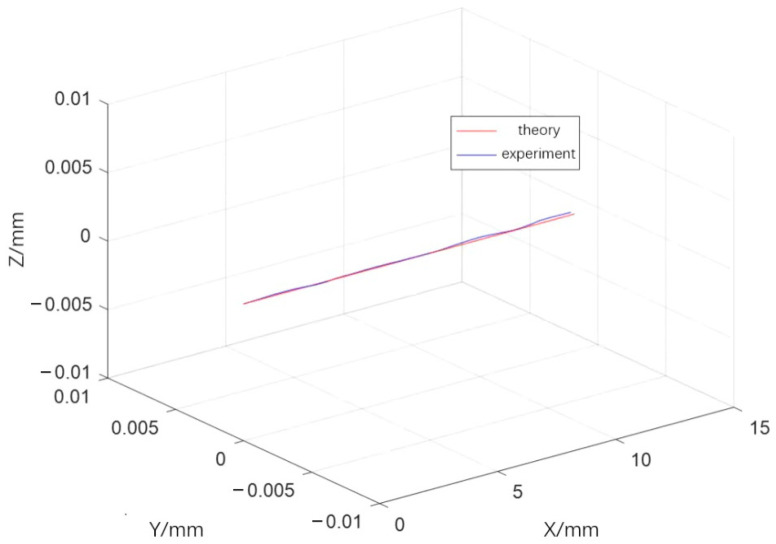
Robot end trajectory and theoretical welding trajectory.

The robot force position hybrid control model was run in Simulink to obtain the actual trajectory of the robot end during the friction stir welding process. This trajectory was compared with the theoretical welding trajectory of the welded plate, as shown in Figure 8.

**Figure 9:**
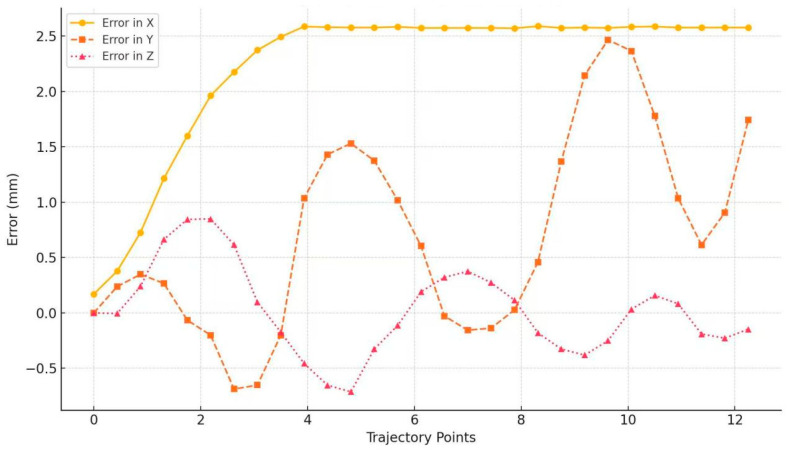
Weld trajectory errors.

In order to more intuitively demonstrate the error between the robot’s end trajectory and the ideal trajectory, as shown in Figure 9, the errors in the X, Y, and Z axis directions of the end trajectory are compared.

Figure 8 shows that the robot end trajectory, as calculated by the force-position hybrid control model, closely approximates the theoretical trajectory. This close approximation is a direct result of employing the enhanced stiffness ellipsoid method, which provides a nuanced understanding of the robot’s stiffness characteristics in various poses. Figure 9 demonstrates that the actual welding trajectory consistently leads the theoretical trajectory in the X direction, with the maximum deviation peaking at approximately 0.020 mm, a precision well within acceptable engineering tolerances. Oscillations in the Y and Z direction errors were observed, yet these are maintained beneath the 0.010 mm threshold, aligning with permissible engineering error margins. 

The actual comparison of the welding seam reveals a significant improvement when using the stiffness ellipsoid method. In the welding seam produced without the method, irregularities and inconsistencies are evident along the seam, such as uneven distribution of material and visible defects. In contrast, the welding seam produced using the stiffness ellipsoid method shows a smooth and uniform appearance with minimal defects. This demonstrates that the stiffness ellipsoid method effectively enhances the precision and quality of the weld seam, ensuring better structural integrity and consistency during the friction stir welding process.

## 4. Conclusions

This study investigates the collaborative control of friction stir welding (FSW) robots and positioners using an improved stiffness ellipsoid method. The research focuses on enhancing the precision and stiffness of robotic welding trajectories through a novel stiffness index and an optimized collaborative motion control model. The proposed method, validated through simulation experiments, demonstrates significant improvements in welding trajectory precision, with errors reduced by up to 60% compared to conventional approaches. Additionally, computational efficiency is enhanced by 40%, highlighting the method’s practicality for industrial applications. These advancements underscore the potential of the improved stiffness ellipsoid method to elevate FSW quality and consistency. Future work should focus on integrating advanced sensors for real-time stiffness adjustment and exploring adaptive control algorithms to further optimize welding performance. Additionally, extending the method to multi-robot systems and complex welding configurations could enhance its applicability and effectiveness.

## Figures and Tables

**Table 1:** Joint stiffness of ZK3000-500 robot.

Joint	Joint Stiffness (N·m/rad)
1	1.14 × 10^7^
2	8.89 × 10^6^
3	8.21 × 10^6^
4	7.44 × 10^6^
5	3.67 × 10^6^
6	2.35 × 10^6^

**Table 2:** Variable comparison table.

Variable Symbol	Variable Meaning	Relevant Formulas and Explanations
K	Cartesian stiffness matrix of the robot end-effector	$K={\left(J^{-1}\right)}^{T}\cdot K_{\theta }\cdot J^{-1}$ (Formula (1)). It is used to describe the stiffness characteristics of the robot end-effector in the Cartesian coordinate system. The elements $k_{ij}$ represent the relationships between forces and displacements in different directions.
$K_{\theta }$	Robot joint stiffness matrix	Involved in establishing the robot joint stiffness model, it is the matrix representation of the robot joint stiffness. It is constructed by obtaining joint stiffness values through static identification experiments.
J	Robot Jacobian matrix	When calculating the Cartesian stiffness matrix, it is used to describe the mapping relationship between the robot joint space and the Cartesian space and is an important part of the formula $K={\left(J^{-1}\right)}^{T}\cdot K_{\theta }\cdot J^{-1}$.
$K_{fd}$	Force–displacement stiffness sub-matrix	It is a sub-matrix of the Cartesian stiffness matrix K (Formula (2)), composed of the first 3 rows and 3 columns of K. It is used to describe the stiffness relationship between forces and linear displacements and is mainly considered when establishing the stiffness ellipsoid.
$K_{f\delta }$	Force–angular displacement stiffness sub-matrix	A sub-matrix of the Cartesian stiffness matrix K (Formula (2)), which describes the stiffness relationship between forces and angular displacements.
$K_{md}$	Torque–displacement stiffness sub-matrix	A sub-matrix of the Cartesian stiffness matrix K (Formula (2)), reflecting the stiffness relationship between torques and linear displacements.
$K_{m\delta }$	Torque–angular displacement stiffness sub-matrix	A sub-matrix of the Cartesian stiffness matrix K (Formula (2)), representing the stiffness relationship between torques and angular displacements.
d	Linear displacement of the robot end-effector along the X, Y, and Z axes	In the formula $F_{d}=K_{fd}\cdot d$ (Formula (3)), it is used to describe the relationship between the linear displacement of the robot end-effector in the Cartesian coordinate system and the force–displacement stiffness sub-matrix.
δ	Angular displacement of the robot end-effector around the X, Y, and Z axes	In the definitions and formulas of relevant stiffness sub-matrices, it is used to describe the relationship between the angular displacement of the robot end-effector and the stiffness sub-matrices. For example, in Formula (2), it reflects its connection with the torque–angular displacement stiffness sub-matrix, etc.
$\lambda_{1}$, $\lambda_{2}$, $\lambda_{3}$	Semi-axis lengths of the stiffness ellipsoid along the x, y, and z axes	In the formula $\frac{x^{2}}{\lambda_{1}}+\frac{y^{2}}{\lambda_{2}}+\frac{z^{2}}{\lambda_{3}}=1$ (Formula (4)), they are used to determine the shape and size of the stiffness ellipsoid. Their values are obtained from the eigenvalues of the real-symmetric matrix $K_{fd}^{T}\cdot K_{fd}$, reflecting the stiffness characteristics of the robot end-effector in different directions.
$\eta_{1}$, $\eta_{2}$, $\eta_{3}$	Eigenvectors corresponding to the eigenvalues of the real-symmetric matrix $K_{fd}^{T}\cdot K_{fd}$	They represent the vector directions of the semi-axes of the stiffness ellipsoid, corresponding to $\lambda_{1}$, $\lambda_{2}$, and $\lambda_{3}$, and determine the orientation of the stiffness ellipsoid in space.
$\alpha_{t}$, $\beta_{t}$, $\gamma_{t}$	Direction cosines of the unit normal vector of the tangent plane of the welding surface	They are used to map the stiffness ellipsoid to the tangent plane of the welding surface to analyze the actual stiffness characteristics of the robot during the welding process.
$\lambda_{t1}$, $\lambda_{t2}$, $\lambda_{t3}$	Stiffness direction quantities related to the new stiffness index	In the formula for constructing the new stiffness index
$V_{f}$	New stiffness index based on the improved stiffness ellipsoid	Used to evaluate the performance of the robot during the friction stir welding process. The formula
$V_{m}$	Objective function of the collaborative motion control model	In the collaborative motion control model, $V_{m}=max\left(V_{f}\right)$ (Formula (7)). The maximum value of the new stiffness index $V_{f}$ is taken as the goal, and the optimal collaborative motion of the robot and the positioner is determined by solving this objective function.
θ	Robot joint angle	In the collaborative motion control model, it is one of the decision variables. It is related to the joint angles of the positioner. For example, $\theta =f\left(\theta_{P1},\theta_{P2}\right)\cdot T_{n}$ (Formula (8)) indicates that the robot joint angle is a function of the positioner joint angles, reflecting the relationship of their collaborative motion.
$\theta_{P1}$, $\theta_{P2}$	Positioner joint angles	They are the decision variables of the collaborative motion control model, used to describe the motion state of the positioner. The robot joint angle θ is a function of them, jointly determining the collaborative motion of the robot and the positioner.
$T_{n}$	Pose information of the weld joint in the robot base coordinate system	In the formula $\theta =f\left(\theta_{P1},\theta_{P2}\right)\cdot T_{n}$ (Formula (8)), it is used to determine the relationship between the robot joint angle and the pose of the weld joint, playing a key role in the collaborative motion control of the robot and the positioner.
$S\left(\theta \right)$	Fitness function of the genetic algorithm	When the genetic algorithm is used to solve the collaborative motion control model, $S\left(\theta \right)=max\left(V_{f}\left(\theta \right)\right)$ (Formula (10)). Based on the new stiffness index $V_{f}$, it is used to evaluate the quality of individuals and guide the genetic algorithm to find the optimal solution.
$\theta_{p}$	Positioner joint angle vector	$\theta_{p}={\left[\begin{array}{cc} \theta_{P1} & \theta_{P2} \end{array}\right]}^{T}$ (Formula (11)). The two joint angles of the positioner are combined into a vector form for convenient calculation and processing in the genetic algorithm and the collaborative motion control model.

**Table 3:** Joint angle and stiffness performance.

Welding Spots	Optimization	$\theta_{p1}({}^{\circ})$	$\theta_{p2}({}^{\circ})$	$\theta_{1}({}^{\circ})$	$\theta_{2}({}^{\circ})$	$\theta_{3}({}^{\circ})$	$\theta_{4}({}^{\circ})$	$\theta_{5}({}^{\circ})$	$\theta_{6}({}^{\circ})$	$V_{f}$
1	before	45.00	0	−1.75	−15.77	48.52	−4.09	37.36	8.13	2476.6
	after	42.46	0.75	−3.01	−14.82	55.02	6.32	37.81	−15.77	2902.4
2	before	45.00	0	−3.24	−25.76	60.05	−10.37	27.21	4.86	2231.7
	after	24.89	6.52	−2.31	−14.49	52.43	−0.53	35.61	−10.51	2792.4
3	before	45.00	0	−11.35	−22.33	65.51	−50.67	25.73	13.27	1847.1
	after	6.67	10.29	−1.21	−14.21	50.15	−5.48	34.41	15.64	3215.8

## Data Availability

The original contributions presented in this study are included in the article. Further inquiries can be directed to the corresponding author.
